# Assessment of Severity of ST-Elevation Myocardial Infarction Using Neutrophil-to-Lymphocyte Ratio and Its Correlation With Thrombolysis in Myocardial Infarction Risk Score

**DOI:** 10.7759/cureus.68877

**Published:** 2024-09-07

**Authors:** Sharan Badiger, Likitha M J, Avinash V Jugati

**Affiliations:** 1 General Medicine, Shri B M Patil Medical College Hospital and Research Centre, BLDE (Deemed to be University), Vijayapura, IND

**Keywords:** angiographic management, angiographic score, myocardial infarction, primary percutaneous coronary intervention (pci), total leucocyte count

## Abstract

Introduction

ST-segment elevation myocardial infarction (STEMI) is an acute presentation of myocardial infarction (MI). It is caused by the complete occlusion of coronary arteries by a thrombus secondary to atherosclerotic plaque formation within these vessels. The Thrombolysis in Myocardial Infarction (TIMI) risk score is a seven-item scale used to categorize patients based on risk and to predict mortality in acute MI patients. The neutrophil-to-lymphocyte ratio (NLR) is an independent assessor of prognosis in cardiovascular diseases; it holds a pivotal role in inflammation and atherosclerotic plaque formation.

Methodology

A hospital-based cross-sectional study was undertaken among 76 patients who had acute MI, out of which 50 patients who had STEMI were included and 26 patients were excluded (13 patients had non-ST-segment elevation myocardial infarction (NSTEMI), four patients had unstable angina, nine patients had arrhythmias). After detailed clinical and laboratory evaluation, NLR was calculated for all 50 patients, and they were distributed into low NLR, intermediate NLR and high NLR groups based on the ratio. Patients then underwent coronary angiography, and their TIMI-STEMI score was calculated and compared with their NLR score. Using SPSS software (IBM Corp., Armonk, NY, USA), the collected data was statistically analyzed.

Results

Fifty patients with acute coronary syndrome (ACS) based on their NLR were distributed into three categories: Category A with low NLR of <1.7 (n=2), Category B with intermediate NLR of 1.7-3 (n=10), and Category C with high NLR of >3 (n=38). In this study, there were more male patients (two in Category A, six in Category B, 28 in Category C), mean age group was 55 +/- 10 (one in Category A, five in Category B, 32 in Category C), the most common presentation was chest pain (two in Category A, nine in Category B, 37 in Category C), the most common risk factor was smoking (two in Category A, six in Category B, 15 in Category C), the angiographic TIMI-STEMI score was calculated and two, six, and two patients in Category A, Category B, Category C respectively had a low score of <4, four and 36 patients in Category B and C respectively had a high score of >4, and there was a significant correlation between high NLR and high TIMI-STEMI score (P = 0.001).

Conclusion

This study infers that a simple bedside parameter like the NLR, which is easily available and affordable, can predict the outcome in STEMI patients and stands on par with conventional angiographic scores.

## Introduction

Worldwide, cardiovascular disease is the main cause of death, with ischemic heart disease accounting for most of the deaths [1]. An extremely high rate of disease severity and mortality is seen in acute presentation leading to ST-segment elevation myocardial infarction (STEMI); this occurs due to a total block in the coronary artery by a thrombus developing from an atherosclerotic plaque within these arteries [2]. Accumulation of oxidized low-density lipoproteins as a result of inflammation is the major cause of such thrombus formation. However, several other complex interactions between inflammatory cytokines and platelets are all responsible for atherosclerotic plaque formation [3].

The Thrombolysis in Myocardial Infarction (TIMI) risk score is a seven-item assessment tool that is used for risk stratification and mortality prediction in STEMI patients; this tool was derived based on multiple trials that were conducted on individuals with STEMI [4]. The risk factors in the STEMI model include age of 65 years or older; at least three risk factors like hyperlipidemia, diabetes, hypertension, smoking, family history; systolic blood pressure less than 100 mmHg on admission; heart rate of more than 100 beats per minute on admission; an admission Killip Heart Failure class II-IV; anterior wall infarction or left bundle branch block; admission weight of less than 67 kg and time to reperfusion therapy more than four hours among patients who receive reperfusion therapy [5].

The neutrophil-to-lymphocyte ratio (NLR) is an independent marker of prognosis in coronary artery disease as it plays a significant role in inflammation and atherosclerotic plaque formation. This NLR is calculated as a simple ratio between the neutrophil and lymphocyte counts measured in peripheral blood [6]. The association between NLR and coronary vascular disease is not clear; certain systemic variables such as oxidative stress, inflammation, and endothelial dysfunction may result in accelerated coronary vascular pathology [7]. The endothelium damage occurs due to the interaction of neutrophils with endothelial tissue, including enhanced neutrophil platelet adhesion and plaque disruption brought on by neutrophil infiltration [8]. Furthermore, by releasing superoxide radicals and proteolytic enzymes, neutrophils lead to increased susceptibility to vulnerable plaque formation. A higher NLR, even with the normal WBC count range, has been associated with cardiovascular events [9].

## Materials and methods

This cross-sectional study was carried out at Shri B M Patil Medical College, Hospital and Research Centre, BLDE (Deemed to be University), Vijayapura, between April 2024 and June 2024. An institutional ethical clearance certificate was obtained (IEC/1095/2023-24). The sample size was calculated to predict a mean with a 95% confidence interval and precision of 0.13 using the t-distribution. The inclusion criteria were all patients with STEMI, and the exclusion criteria were patients with congenital heart disease, heart transplants, those who have undergone prior coronary revascularization procedures, the presence of non-STEMI (NSTEMI), unstable angina, and arrhythmias. Detailed history, including demographic data, risk factors, and presenting complaints, were noted, and clinical examination was carried out, which included weight, height, BMI, blood pressure measurement and systemic examination. Patients underwent all routine blood investigations, including complete blood count, lipid profile, hemoglobin A1C (HbA1C), and renal function tests. Additionally, an electrocardiogram (echo), 2D echo, and coronary angiography were performed. A total of 76 patients with STEMI were enrolled; 50 patients who fulfilled the inclusion criteria were recruited into this study, and the remaining 26 patients were excluded with the following exclusion criteria: 13 patients had NSTEMI, four patients had unstable angina, and nine patients had arrhythmias. In the 50 patients fulfilling the inclusion criteria, prior informed and written consent was obtained, and the NLR upon presentation was calculated; based on this they were distributed into three categories: Category A with low NLR (<1.7), Category B with intermediate NLR (1.7-3.0), Category C with high NLR (>3.0) [10]. This was compared with the TIMI-STEMI angiographic score, where a score of <4 was considered low and a score of >4 was considered high.

Statistical analysis

All the obtained data was analyzed using Statistical Package for Social Science SPSS (version 20) software (IBM Corp, Armonk, NY), and data was represented using graphs, counts and percentages, mean, and standard deviation. The data with continuous variables and those that are regularly distributed were compared using an independent t-test; for categorical variables, the Chi-square test was used. Mann-Whitney U test was used to test variables that were not regularly distributed. All statistical tests were performed two-tailed, and results were considered statistically significant if the P value <0.05.

## Results

**Figure 1:**
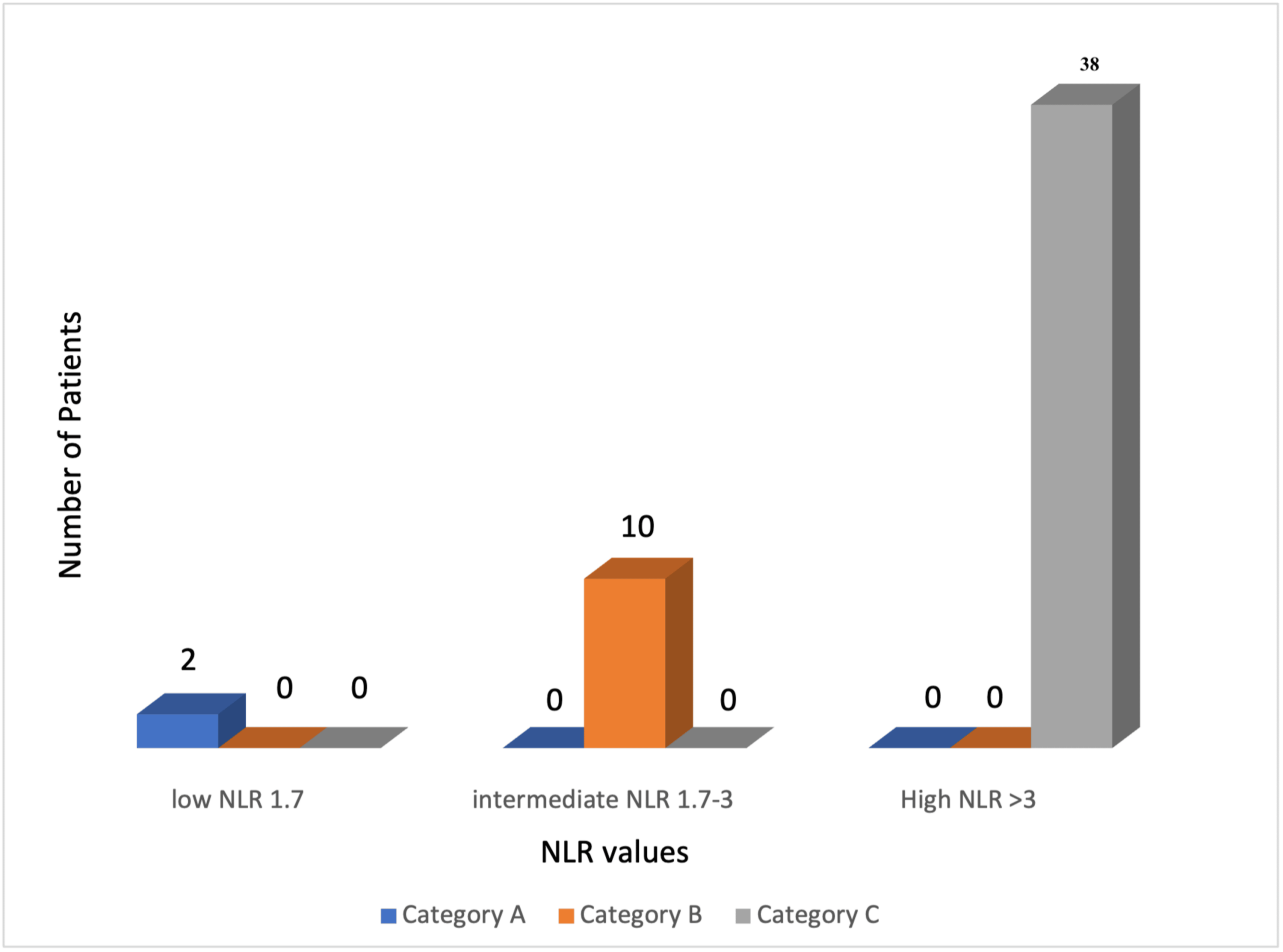
Patient distribution based on Neutrophil-to-Lymphocyte Ratio (NLR)

Fifty patients with acute coronary syndrome (ACS) based on their NLR were distributed into three categories, NLR of <1.7 as category A (n=2; 4%), intermediate NLR of 1.7 to 3 as category B (n=10; 20%) and high NLR of >3 as category C (n=38; 76%) as depicted in Figure 1. The mean age in study sample of 50 patients was 55+/- 10 years. One patient (n=1; 50%) of two males in category A (n=2; 100%), two patients (n=2; 33.3%) of six male patients, and one patient (n=1; 25%) out of four female patients in category B (n=10; 100%), and 10 patients (n=10; 35.7%) of 28 male patients, six patients (n=6; 60%) of 10 female patients in category C (n=38; 100%) belonged to a common age group of 51 to 60 years as shown in Table 1, indicating that ACS is disease of elderly and more prevalent in male gender. This could be due to underlying atherosclerotic pathology, the risk of which increases with increasing age. 

**Table 1:** Demographic and Clinical Data

Demographic data	Category A (n=2)		Category B (n=10)		Category C (n=38)		Chi Square	P value
Age Group	Male (n=2)	Female (n=0)	Male (n=6)	Females (n=4)	Male (n=28)	Female (n=10)	0.23	0.91
21-30 years	0(00.0%)	0(00.0%)	0(00.0%)	0(00.0%)	0(00.0%)	0(00.0%)		
31-40 years	0(00.0%)	0(00.0%)	0(00.0%)	0(00.0%)	2(7.14%)	0(00.0%)		
41-50 years	0(00.0%)	0(00.0%)	1(16.6%)	0(00.0%)	3(10.7%)	0(00.0%)		
51-60 years	1(50.0%)	0(00.0%)	2(33.3%)	1(25.0%)	10(35.7%)	6(60.0%)		
61-70 years	1(50.0%)	0(00.0%)	2(33.3%)	3(75.0%)	8(28.0%)	4(40.0%)		
>70 years	0(00.0%)	0(00.0%)	1(16.6%)	0(00.0%)	5(17.8%)	0(00.0%)		
Clinical Data								
Chest pain	2(100%)	0(00.0%)	6(100%)	3(75.0%)	28(100%)	9(90.0%)	0.152	0.767
Dyspnea	1(50.0%)	0(00.0%)	1(16.6%)	3(75.0%)	4(14.2%)	3(30.0%)	0.616	0.968
Palpitation	0(00.0%)	0(00.0%)	2(33.3%)	1(25.0%)	6(21.4%)	1(10.0%)	0.733	0.965
Smoking	2(100%)	0(00.0%)	6(100%)	0(00.0%)	20(71.4%)	0(00.0%)	0.107	0.688
Hypertension	1(50.0%)	0(00.0%)	3(50.0%)	3(75.0%)	10(35.7%)	5(50.0%)	0.787	0.91
Diabetes	1(50.0%)	0(00.0%)	2(33.3%)	3(75.0%)	8(28.0%)	6(60.0%)	0.111	0.91

The most common presenting symptom was chest pain which was seen in 48 out of 50 patients (n=48; 96%), in category A; two out of two male patients (n=2; 100%), in category B; six of six male patients (n=6; 100%), three of four female patients (n=3; 75%), and in Category C; 28 of 28 male patients (n=28; 100%) and nine of 10 female patients (n=9; 90%) had chest pain. Other symptoms included breathlessness which was seen in 12 out of 50 patients (n=12; 24%) of which there was one of two male patients (n=1; 50%) in Category A, one of six male patients (n=1; 16.6%), three of four female patients (n=3; 75%) in Category B and four of 28 male patients (n=4; 14.2%) and three of 10 female patients (n=3; 30%) in category C. Palpitations was seen in total 10 out of 50 patients (n=10; 20%) out of which no patients in category A; (n=0; 0%), two of six male patients (n=2; 33.3%), one of four female patients (n=1; 25%) in category B and six of 28 male patients (n=6; 21.4%) and one of 10 female patients (n=1; 10%) in category C. Out of all the risk factors smoking was found to be most common, it was seen in 28 out of 50 patients (n=28; 56%) of which two of two male patients (n=2; 100%) in category A, six of six male patients (n=6; 100%) in category B, and 20 of 28 male patients (n=20; 71.4%) in category C, had history of smoking. Hypertension was the next common risk factor seen in 22 out of 50 patients (n=22; 44%) in category A; one of two male patients (n=1; 50%) in category B; three of six male patients (n=3; 50%) three of four female patients (n=3; 75%) and in category C, 10 of 28 male patients (n=10; 35.7%) and five of 10 female patients (n=5; 50%) had hypertension. Diabetes was present in 20 out of 50 patients (n=20; 40%), in category A, one of two male patients (n=1; 50%) in category B, two of six male patients (n=2; 33.3%) three of four female patients (n=3; 75%) and in category C; eight of 28 male patients (n=8; 28%), six of 10 female patients (n=6; 60%). In this study, most patients presented with chest pain, which is the most common presenting symptom of myocardial infarction; also, these results demonstrate that most of the patients had conventional risk factors like smoking, hypertension and diabetes, all of which are modifiable with changes in the lifestyle. The demographic and clinical data of all 50 patients is summarised in Table 1, and the hemodynamic and laboratory data is summarised in Table 2.

**Table 2:** Hemodynamic and Laboratory Data

Hemodynamic and laboratory data	Reference Range	Mean	Standard deviation (SD)	Mean	Standard deviation (SD)	Mean	Standard deviation (SD)	Mann-Whitney U	P Value
Pulse rate (beats per minute)	80-100	31.3	9.786	51.20	12.558	96.54	18.010	164.000	0.04
Systolic blood pressure (mmHg)	90-120	83.4	11.898	43.78	0.576	19.60	1.673	117.000	0.03
Haemoglobin (g%)	13-17	13.1020	2.12608	7.40	18.50	15.976	33.457	158.000	0.01
Total Leucocyte Count (10^^^3/ml)	4-10	3600.0	21290.00	11719.6	4091.55577	4598.9	5.677	176.000	0.015
Neutrophil count (cells/ml))	2500-7000	11719.6800	4091.55577	3600.00	21290.00	156.8	32.923	184.000	0.045
Lymphocyte count (cells/ml)	1000-4800	9300.6	3973.25253	2131.00	18984.00	29.20	1.678	119.000	0.03
Eosinophil count (cells/ml)	30-350	1813.7	650.51745	240.00	3553.00	82.47	28.245	178.000	0.09
Platelet count (10^^^3/ml)	150-410	2.8400	3.59342	.00	23.00	103.80	27.898	156.000	0.10
Blood Urea (mg/dl)	19-43	264.56	67.27712	148.00	446.00	413.34	59.973	196.000	0.10
Serum Creatinine (mg/dl)	0.4-1.1	26.900	10.95864	13.00	76.00	475.18	48.778	188.000	0.20
Total Cholesterol (mg/dl)	<200	178.34	21.657	173.62	20.659	174.9	32.92	196.000	0.985
Triglycerides (mg/dl)	<150	138.97	78.433	118.72	28.89	141.12	84.56	190.000	0.90
Troponin-I (pg/ml)	<11.6	0.8780	0.23586	0.50	1.60	136.70	4.775	111.000	0.01

The angiographic data of 50 patients was analysed and 24 of 50 patients (n=24; 48%) had single vessel disease (SVD), 16 patients (n=16; 32%) had double vessel disease (DVD) and 10 patients (n=10; 20%) had triple vessel disease (TVD). Out of 24 patients with SVD two patients belonged to category A (n=2; 8.3%), two patients belonged to category B (n=2; 8.3%) and 20 patients belonged to category C (n=20; 83.3%). In 16 patients with DVD there were no patients belonging to category A, six patients belonging to category B (n=6; 37.5%) and 10 patients belonging to category C (n=10; 62.5%). Out of 10 patients with TVD, no patients belonged to Category A, two patients belonged to category B (n=2; 20%) and eight patients belonged to category C (n=8; 80%). Total number of patients in category A were two (n=2; 4%), in category B were 10 (n=10; 20%) and in category C were 38 (n=38; 76%) out of the 50 patients with SVD, DVD and TVD. The angiographic TIMI-STEMI score was calculated in 50 patients of all three categories; 10 patients had low TIMI-STEMI score of <4, and 40 patients had high TIMI-STEMI score of more than four. Two out of two patients in category A (n=2; 100%), six out of 10 patients in category B (n=6; 60%) and two out of 38 patients in category C (n=2; 5.6%) had low TIMI-STEMI score of <4. No patients in category A (n=0; 0%), four out of 10 patients in Category B (n=4; 40%) and 36 out of 38 patients in category C (n=36; 94.7%) had high TIMI-STEMI score of >4. This was compared with the NLR ratio, and there was a significant correlation between the high NLR (Category C) and high TIMI-STEMI score (p=0.001) (Table 3).

**Table 3:** Correlation between Neutrophil-to-Lymphocyte Ratio (NLR) and angiographic TIMI-STEMI Score *Significant value (P<0.05) STEMI: ST-segment elevation myocardial infarction; TIMI: Thrombolysis in Myocardial Infarction

Correlation b/w TIMI-STEMI score and NLR	Low TIMI-STEMI Score <4	High TIMI-STEMI Score >4	Chi Square Value	P value
Low NLR <1.7 (Category A n=2)	2 (100%)	0 (0.00%)	23.13	*0.001
Intermediate NLR 1.7-3 (Category B n=10)	6 (60.0 %)	4 (40.0%)		
High NLR > 3 (Category C n=38)	2 (5.26%)	36 (94.7%)		

## Discussion

The association of NLR with many cardiovascular diseases has been studied before; however, very few attempts have been made to study its correlation with angiographic TIMI-STEMI score. In this study, the most common age group observed was 55 +/-10 years, which was similar to a study done by Jingyu et al. in Beijing, China, from 2002 to 2005; in their study, they included 692 patients with STEMI with most common age group of 55 to 65 years and they used NLR to predict the long term mortality in these patients, they concluded that patients with high NLR of >4 had a four-fold increase in all-cause mortality [11]. The commonest risk factor, as noted in this study, was smoking, followed by hypertension and diabetes; a similar association was noted in one such study conducted by Ulas et al. in Turkey in 2021; in this study they aimed to find out the prognostic value of several blood parameters including NLR, WBC, mean platelet volume (MVP) on 259 patients with NSTEMI and they showed that most common risk factor was hypertension followed by smoking and diabetes [12].

In this study, there was a wide range of total leucocyte count, with the least count of 3,600 cells/cumm to a maximum count of 21,690 cells/cumm. However, patients with less total leucocyte count still showed intermediate to high NLR. Another cohort study done by Rostami et al. in October 2021 included 200 STEMI patients who then underwent primary percutaneous coronary intervention (PCI), and they utilised NLR and systemic inflammatory immunologic index (SII) to predict the post-procedural no-reflow; this study showed that WBC ranged from 10,000 cells/cumm to 14,000 cells/cumm with most patients with higher WBC counts in high NLR group [13]. Based on coronary angiography it was observed that 24 of 50 of our patients (n=24; 48%) had single vessel disease, 16 of 50 patients (n=16; 32%) had double vessel disease, and 10 of 50 patients (n=10; 20%) had triple vessel disease. In 16 of 50 patients with double vessel disease six of them belonged to category B with intermediate NLR between 1.7 to 3, in another study conducted by Pan et al. in 2014 who investigated the NLR potential in predicting mortality and coronary flow showed that 167 out of 636 patients in low intermediate NLR group had more than one coronary vessel involvement [14].

In this study, 10 of 50 patients (n=10; 20%) of patients had an intermediate NLR of 1.7-3, and 38 of 50 patients (n=38; 78%) of patients had a high NLR of >3. When the angiographic TIMI-STEMI score was calculated for these 50 patients, it was noted that 40 out of 50 patients (n=40; 80%) had high TIMI-STEMI score of more than 4. Out of 40 patients with high TIMI-STEMI score, 36 of them (n=36; 94.7%) belonged to the high NLR category with a statistically significant correlation between high NLR and high TIMI-STEMI score (P=0.001), inferring that NLR ratio is on par with angiographic scores in predicting the severity of the acute myocardial infarction. Similar conclusions were drawn by another study done by Erkol et al. in 2014; in this retrospective study including 1,625 acute STEMI patients, they aimed to study whether spontaneous infarct-related artery patency was associated with NLR, they concluded that NLR was significantly lower in patients with patent infarct-related artery (P< 0.001), it was also observed that in patients with TIMI-STEMI score ≥4 the NLR was more, than in patients with TIMI-STEMI score of <4 (p=<0.001) [15]. The use of Internet of Medical Things (IoMT) can now provide timely healthcare in remote places, which helps in early diagnosis and management of MI cases, thereby reducing overall disease morbidity and mortality. In this era of telemedicine and rapidly evolving medical technology, the field of IoMT is rapidly evolving [16].

Limitation

In this study, the patient sample has been taken from a single tertiary care centre; a much larger sample size from multiple centers will give better clarity about the potentiality of NLR to assess the severity of ACS.

## Conclusions

This study demonstrated the significant correlation between NLR and TIMI-STEMI score, suggesting that ACS patients with a high burden of inflammation (indicated by high NLR) are at greater risk of developing severe atherosclerosis, leading to a more severe form of ACS. When compared with their angiographic TIMI-STEMI score, the study also showed that patients with high NLR had a higher risk of thrombus formation and transmural infarction (TIMI-STEMI score >4). The NLR is a practical, inexpensive, and consistent bio-marker in assessing the severity of STEMI patients. This simple bedside clinical marker, when integrated with an established clinical risk assessment score like the TIMI-STEMI score, can give a more accurate prognostication of a patient's condition. The significant findings from our study hope to offer valuable guidance for future studies on larger study groups.
